# Protein protein interactions, evolutionary rate, abundance and age

**DOI:** 10.1186/1471-2105-7-128

**Published:** 2006-03-13

**Authors:** Ramazan Saeed, Charlotte M Deane

**Affiliations:** 1 Department of Statistics, 1 South Parks Road, Oxford OX1 3TG, UK

## Abstract

**Background:**

Does a relationship exist between a protein's evolutionary rate and its number of interactions? This relationship has been put forward many times, based on a biological premise that a highly interacting protein will be more restricted in its sequence changes. However, to date several studies have voiced conflicting views on the presence or absence of such a relationship.

**Results:**

Here we perform a large scale study over multiple data sets in order to demonstrate that the major reason for conflict between previous studies is the use of different but overlapping datasets. We show that lack of correlation, between evolutionary rate and number of interactions in a data set is related to the error rate. We also demonstrate that the correlation is not an artifact of the underlying distributions of evolutionary distance and interactions and is therefore likely to be biologically relevant. Further to this, we consider the claim that the dependence is due to gene expression levels and find some supporting evidence. A strong and positive correlation between the number of interactions and the age of a protein is also observed and we show this relationship is independent of expression levels.

**Conclusion:**

A correlation between number of interactions and evolutionary rate is observed but is dependent on the accuracy of the dataset being used. However it appears that the number of interactions a protein participates in depends more on the age of the protein than the rate at which it changes.

## Background

It has been suggested many times that the rate at which a protein evolves decreases with the number of physical interactions it participates in [3-6]. The intuition behind this idea is that proteins with a greater fraction of amino acid residues playing an essential role will, on the whole, evolve slower then those with a small ratio of such crucial residues. Thus highly interacting proteins will evolve at a slower rate. A recent study by Fraser et al (2002) demonstrated the negative correlation, which this theory would suggest between protein-protein interactions and evolutionary rate. The negative correlation was determined by estimating the evolutionary distance between orthologous proteins from yeast *Saccharomyces cerevisiae *and the nematode worm *Caenorhabditis elegans*. Using interaction data from studies conducted by Uetz and "core data" from Ito it was shown that yeast proteins possessing a large number of interacting partners evolve slower then those that have fewer interacting partners. However this relationship proved to be contentious, and the observation was challenged by Jordan et al (2003) on the basis that a correlation between a proteins evolutionary rate and number of interactions arises only because a few highly interacting proteins evolved more slowly. In Jordan's study a smaller set of interactions were considered (The MIPS dataset) and a different measure of evolutionary distance was adopted, using the distance between two yeast species *S cerevisiae *and *S. pombe *[*7*]. Although one would expect that such a comparison would result in an increase in the strength of the relationship, as more orthologs could be found between two closely related species and evolutionary distance could be estimated with greater precision, this was shown not to be the case and only a very weak correlation was detected. This finding was immediately rebutted by Fraser et al (2003) who claimed that the dataset used in the study conducted by Jordan et al (2003) was too small. They also stated that the method used to obtain evolutionary distance resulted in low confidence data and hence the lack of any correlation [8]. Bloom et al (2003) then demonstrated that the presence of any correlation between evolutionary rate and number of interactions was dependent on the dataset that was used [9].

Experimental methods used to obtain interaction datasets include the Yeast two-hybrid (Y2H) assay and Mass spectrometry (MS) of purified complexes. In the Y2H method, pairs of proteins to be tested for interaction are expressed as fusion proteins in yeast (hybrids): one protein is fused to a DNA-binding domain, the other to a transcriptional activator domain. Any interaction between them is detected by the observation of a reporter gene which results from the formation of a transcription factor [10]. It is an in vivo technique and both transient and unstable interactions can be detected. It is independent of native protein expression levels and has a fine resolution enabling interaction mapping between proteins.

Drawbacks include the fact that only two proteins are tested at a time (no cooperative binding is detected) and the binding takes place in the nucleus. Consequently many proteins are not in their native compartment and interactions between proteins are unrelated to the physiological setting. Auto activation of the transcription factor can also occur and the fusion process may malform the hybrids.

In the Mass Spectrometry of purified complexes, individual proteins are tagged and used as 'hooks' to biochemically purify whole protein complexes. These are then separated and their components identified by mass spectrometry. Two protocols are widely used: tandem affinity purification (TAP) and high-throughput mass-spectrometric protein complex identification (HMS-PCI). In this method several members of a complex can be tagged, giving an internal check for consistency; and it detects real complexes in physiological settings. Drawbacks include the fact that some complexes that are not present under the given conditions may be missed, tagging may disturb complex formation, and loosely associated components may be washed off during purification [11].

Previous studies have shown that data generated from such large scale experiments have varying error rates and that the number of overlapping interactions is low [12-14]. Explanations for this include that: the methods have not reached saturation point; different methods produce a large number of false positives; and some methods may have difficulties detecting certain types of interactions. Studies that have assessed the reliability of these datasets have uniformly acknowledged that data obtained from the Y2H studies contain high error rates and protein complex purification methods have a slightly higher level of accuracy. There remains a lack of analysis on the error rates within protein interactions databases that have gathered interaction data from numerous sources.

A further complication was highlighted by Bloom. Some of the experimental methods were shown to be biased towards counting more interactions for abundant proteins [9]. This is not a universally accepted conclusion and Fraser et al insisted that it is entirely possible that this manner of relationship between expression levels and number of interactions is an intrinsic characteristic of yeast rather than any experimental bias [15].

This link between abundance and experimental methods is of particular interest as it is known that highly abundant proteins evolve slower [16]. Bloom et al. demonstrated a strong negative correlation between the rate of evolution and the abundance of a protein. This reported correlation was far stronger than the correlation between evolutionary rate and connectivity [9]. Bloom et al (2003) assert that the relationship between expression levels and connectivity was responsible for the negative correlation between evolutionary rate and connectivity.

Some of the studies that observed correlations between connectivity and rate of change did not control for the abundance levels of proteins [17,18]. It is clear that there is a strong relationship between connectivity and the expression level of a protein in individual experimental datasets [9]. Whether this relationship is still observed in accumulative interaction datasets (sets containing interaction data from multiple experimental sources) has yet to be investigated.

Wuchty (2004) examined the relationship between protein essentiality, connectivity, rate of change and conservation [18]. A negative correlation was found between rate of change and connectivity. However using a novel method to quantify the conservation of a protein, Excess Retention (ER), it was observed that both essentiality and connectivity correlated better with ER than with evolutionary rate. Unfortunately all these contradicting studies conducted their analyses on different datasets. Studies in which a correlation was observed used different data to that of studies where no correlation was observed. This leads to the possibility that the discrepancies between studies arise from the different protein interactions datasets, particularly if the errors in these datasets vary.

Here we analyse six widely accessible protein-protein interaction databases for the yeast *S. cerevisiae*. We calculated the evolutionary distance to the *Mus musculus *and the yeast species *S. paradoxus *using varying methods and then examined the resulting correlations. We considered the overlap of interactions in all the datasets, and calculated three separate measures for the accuracy of each dataset.

In general where no correlation was found in a dataset, it was because the dataset had a large number of interactions derived from experimentally inaccurate methods. Datasets with a high overlap from more robust experimental methods showed an obvious relationship. We show that where a negative correlation is observed it is not due to the simple combination of the distribution of evolutionary rate and number of interactions, but because of some underlying biological factor.

We also examine the impact of gene expression levels and protein age on our observed correlations. In line with previous findings we show that, in all datasets, highly expressed proteins evolve slower. In datasets where we observed a correlation between the number of interactions and evolutionary rate we also find that proteins that are highly expressed are also highly connected. We also find that older proteins possess a larger number of interactions and this is independent of protein expression levels.

## Methods

### Data

The following Protein-Protein interaction datasets were used in this study: DIP, MIPS, BIND, GRID, MINT, INTACT [19-24]. A self interaction was counted as one interaction against the interacting protein. Duplicates and duplicates by virtue of inversion were removed from the interactions sets.

Protein sequences for interaction sets, where possible were downloaded from the dataset's corresponding website. In all other cases they were obtained from either SGD or UniProt depending on the annotation of proteins [25,26]. Not all interacting proteins could be assigned protein sequences.

The MIPS dataset contained two types of interactions, physical and genetic. Physical interactions are those ascertained from Y2H studies and purified complexes while genetic interactions were obtained from suppression mutation and synthetic lethality tests. These two types were treated as two different sets.

The Intact dataset housed a *small *set of interactions, that contained results from small to medium scale experiments as well as the results from the four large scale studies [27-30]. These were treated as individual sets.

### Evolutionary distance

Best Reciprocal Hit (BRH) orthologs in each interaction set were found using the BLASTP program [31] and comparing to the entire proteome of either *Mus musculus *[1] or *Saccharomyces paradoxus *[2]. The evolutionary rate was estimated using two methods. The first [32] required us to numerically solve the equation *q *= [*ln*(1 + 2*d*)]/2d, where *q *is the proportion of identical sites between aligned sequences and *d *is the evolutionary distance. The second method used the gamma distance correction [7]*d = α *[(1 - *p*)^-1/*α *^- 1] where *d *is the evolutionary distance between two protein sequences, *p *is the number of different residues, and *α *is the estimated gamma shape parameter, *α *= 1.53 [33].

### Randomisation

The randomisation test was conducted by systematically selecting a protein in a dataset, and assigning it an evolutionary rate by sampling the distribution of evolutionary distances. These random rates were then plotted against the number of interactions. 100 sets were generated, their correlation coefficients were calculated and compared to the correlation coefficient of the original experimental set.

### Overlap

We universalised the labelling of all protein interactors in order to overcome the use of different notations to mark proteins. This was done by matching the sequence of each protein interactor to every sequence in the yeast genome (entire genome downloaded from SGD). Only 100% sequence matches were reannotated with the GenBank id, (GI Code). Protein attrition was no more then 5% in all datasets.

### Abundance

Gene expression level data was taken from the Young lab [34].

### Error rates

We used three methods to assess the accuracy of the different interaction data sets.

The first was the expression profile reliability (EPR) index [14]. The EPR index is calculated using an online server [19]. An expression based distance score is calculated for all interacting protein pairs in a set.

The resulting distribution of distance scores is compared to the distance score distributions of standard interacting and noninteracting sets. The comparison yields the approximate percentage of true interactions in the set.

The second error rate indicator, the Reference Index, involves comparing each interaction against a reference set, following work done by Von Mering et al (2002). The reference set used was the DIP_Core dataset which is considered to be a good interaction set [19]. This dataset contains protein interactions that have been computationally verified or observed in more than one large-scale experiment or those that come from small scale experiments. The percentage of interactions, from the dataset of interest, present in the DIP_Core dataset is taken as an indicator of the reliability of that set.

The third estimator of error rate was the percentage of interacting proteins that shared the same subcellular localisation. In an extension to the logic that interacting proteins would share similar functional roles, it is also possible to say that they would share similar subcellular compartments [35]. The number of interactions in which both interacting partners share the same compartment is used to give a measure of error within an interaction dataset. The subcellular localisations of yeast proteins into 19 compartmental categories is available [36]. The Localisation Similarity (LS) index was the fraction of interactions in which both proteins were from the same compartmental category.

In order to quantify the effect of error rates on each dataset, we ranked the dataset according to each measure of error. 1 being the highest rank and 0 if no error measure existed for the dataset. By calculating the mean rank of each dataset we obtain a consensus measure of error.

### Evolutionary excess retention

To estimate the age of a protein we calculated the Evolutionary Excess Retention (ER), previously used as a measure for conservation of a protein [18]. The ER is a value that depicts the propensity of a protein to have orthologs in other fully sequenced genomes. It should be noted that ER does not estimate the exact age of a protein and is not necessarily correct for all proteins, as it does not identify gene loss or consider horizontal gene transfer. Therefore proteins that have a high ER value are most likely to be old but proteins with a low ER value may not necessarily be new.

We measured for orthologs in *S. cerevisiae *and *H. sapiens, D. melanogaster, C. elegans, M. musculus *and *A. thaliana*. Orthologs were taken from the InParanoid database [37] and we only used those core pairs of each cluster that had a confidence of 100%.

## Results

### Evolutionary rate

The number of proteins and their respective interactions varied in each dataset as shown in Table 1. The largest set according to number of proteins, was GRID followed by the DIP_Full set. The number of proteins in a set does not necessarily dictate the total number of interactions in that set. The DIP_Full set contains 15,481 interactions while the BIND dataset, with only ~5% less proteins, possesses approximately half that number of interactions. The smallest dataset was INTACT_Small, which consists of data solely from a few small scale experiments.

Due to nomenclature and curation errors a few proteins without sequence data remained (Table 1). This resulted in the loss of some interactions from our final analysis. The BIND dataset was particularly affected.

**Figure 1:**
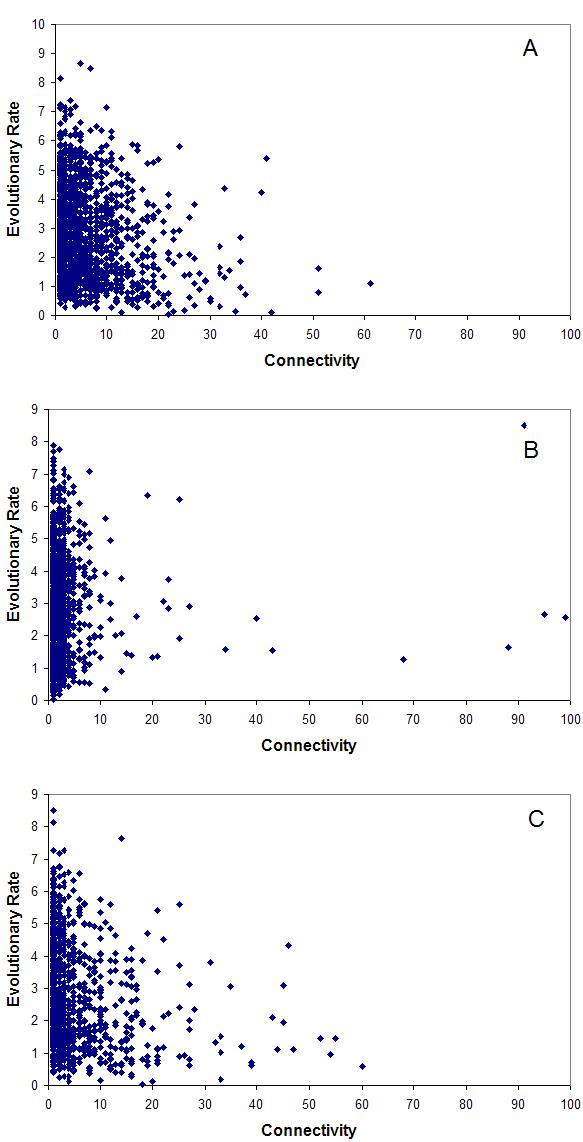
**Observed Correlations**. The observed correlations between interactions and evolutionary rate in three datasets. Correlations were statistically quantified using Spearman's rank correlation coefficient. **A **The correlation observed in the DIP_Core dataset *ρ*: -0.16819. Grishin's method was used to obtain evolutionary rate. **B **No correlation was observed in the INTACT_ITO dataset *ρ*: 0.03 **C **The INTACT_HO dataset showed our strongest correlation *ρ*: -0.227. Proteins with more than 100 interactions are not shown on the figures to aid clarity.

Figure 1A shows the correlation between evolutionary rate and number of interactions for the DIP_Core dataset. The Spearman's rank correlation coefficient returned by the DIP_Core data was -0.1682 with a P-value of 1.29*e*^-11 ^indicating the statistical significance of this weak correlation. This negative correlation suggests that proteins with a larger number of interactions tend to evolve slower.

**Figure 2:**
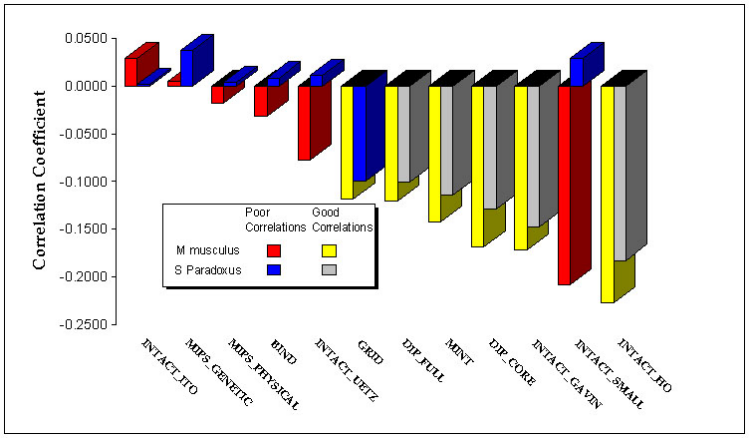
**Dataset Correlations**. The bar graph shows the Spearman's rank correlation coefficients observed between number of interactions and evolutionary rate. Evolutionary rate was estimated using two sets of orthologs, from *M musculus *and *S paradoxus*. Good correlations all had low P-values (< 0.03) while the poor correlations had high P-values > 0.03. Where P-values were significant, correlations using *S paradoxus *orthologs were slightly weaker, yet the general magnitude followed the same trend shown by the correlations obtained using *M musculus *orthologs.

Varying the model used to estimate evolutionary rate between Grishin's and Ota's method had little effect. The Spearman's rank correlations for all the datasets, using Grishin's method for evolutionary rate, are shown in Table 1. None of the sets returned very strong correlations. The strongest correlation was observed in the INTACT_Ho dataset, a fully experimental set obtained by complex purification. INTACT_Ito, a purely Y2H dataset shows no correlation at all (Spearman's *ρ*: 0.03, P-value: 0.8905). The INTACT_SMALL dataset has a very small number of interactions and it returned the second strongest correlation, however its statistical significance was low (P-value: 0.0508). Figure 2 shows all the datasets ranked by their correlation values. The five datasets with the worst correlations, (Spearman's *ρ *> -0.1) returned high P-Values (> 0.01).

We also estimated the evolutionary rate of proteins by finding orthologs in the species *S. paradoxus*. Using a more closely related species to estimate evolutionary rate resulted in a greater number of orthologs. In all but the BIND dataset, we found orthologs for over 90% of the interacting proteins. Figure 2 shows the Spearman's rank corrolations for evolutionary rate estimated from *S. paradoxus *orthologs using Grishin's method, against number of interactions.

### Error rates and overlap

To obtain the error rates in the datasets we used three indicators of correctness. The EPR index, Reference index and the localisation similarity (LS) index. All three error rates for the datasets are listed in Table 2. Annotational issues with the BIND dataset resulted in an inability to calculate its EPR or LS index. Approximately 40% of the interactions in the BIND dataset had at least one partner protein, for which no expression information or localisation categorisation could be found.

Not all error rate measures agree for particular datasets. For example, the MIPS_Genetic dataset has a high EPR Index. This would indicate a large number of true positives, yet when comparing to a reference set the overlap is only 4.08% which is a low value compared to other datasets. The corresponding localisation similarity is at 27%, which is on the lower end of the LS index spectrum.

The INTACT_SMALL dataset possessed a strong correlation between connectivity when *cerevisiae-musculus *orthologs were used yet the statistical significance of this correlation was very low. Furthermore it returns an abnormally high EPR Index. This is because of the statistical nature of both tests. The small size of the INTACT_SMALL dataset makes the significance of any results highly dubious. The DIP_Core dataset, our reference dataset, has one of the lowest error rates. It possesses the second highest EPR Index and also a high LS index. The ITO and UETZ datasets (both large scale Y2H experiments) have high error rates, corroborating previous error rate analysis [13].

**Figure 3:**
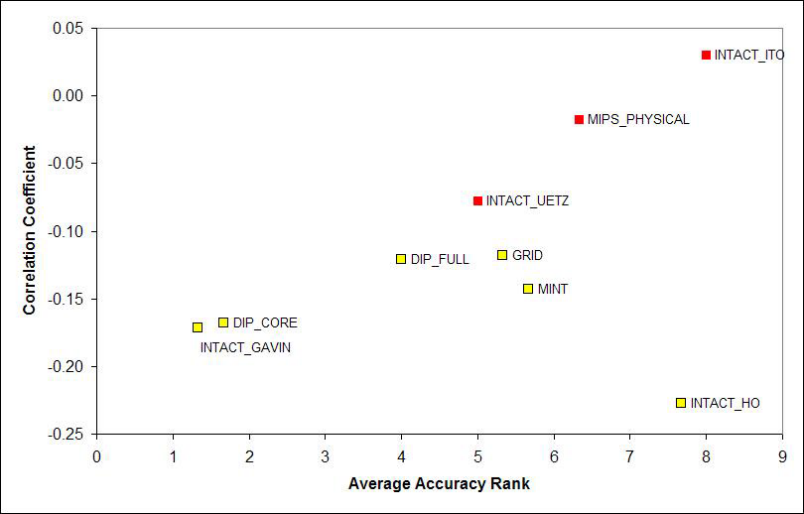
**Error Rates in dataset**. The Spearman's rank correlation coefficient, obtained using *M musculus *orthologs, is shown against a consensus measure for error rates. The consensus measure is calculated by averaging the rank of datasets for the three measures of error. Correlation coefficients marked in yellow indicate significant correlations, and the red markers indicate low statistical significance (Figure 2). Datasets that demonstrate stronger correlations between connectivity and evolutionary rate are more accurate whereas datasets that show no correlation are found to be less accurate. The notable exception here is the INTACT_HO dataset, and this may arise from experimental bias.

To obtain a consensus measure of the error rates, we calculated the mean accuracy rank for each dataset, based upon its rank in each measure. Figure 3 shows a graph of the consensus measure for all the datasets with the exception of the BIND, INTACT_SMALL and MIPS_GENETIC datasets. These three datasets are excluded as accurate error rates cannot be calculated for them.

The overlaps between the accumulative datasets (sets containing data from many experimental sources) and the four major experimental studies were also calculated. Table 3 shows the percentage of interactions from the five INTACT datasets (the four major experimental studies and a fifth set containing interaction from small scale experiments) and data from the remaining databases. An interesting observation is that all the accumulative datasets that had no correlation i.e. both the MIPS datasets and the BIND dataset, have very little overlap with affinity purification datasets. They do however contain a substantial number of interactions obtained from the large scale Y2H studies.

### Randomisation

A randomisation test was carried out to demonstrate that the observed negative correlation was not just a simple combination of the distribution of evolutionary rate and number of interactions. The randomisation study was conducted on those datasets that returned a significant negative correlation, to clarify that it was in fact a biological factor that was responsible for the correlation.

**Figure 4:**
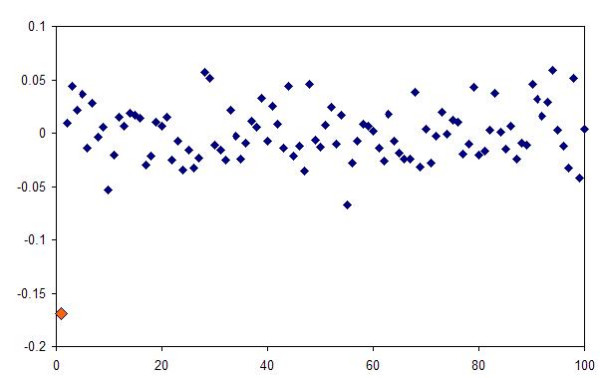
**Randomisation**. Results of the randomisation study on the DIP_Core dataset. The first marker (red) represents the correlation of the original dataset. All other markers show the correlations of the 100 randomly sampled datasets.

Figure 4 shows the results from a randomisation study conducted on the DIP_Core dataset. It can be seen that the correlation of the DIP_Core dataset stands out amongst the correlations of the randomised datasets. This was also the case for all datasets that showed a significant correlation.

### Abundance

We consistently found a negative correlation between evolutionary rate and the expression level of a protein (Spearman's *ρ *~ -0.4). In other words proteins that were more abundant in the cell tended to evolve at a slower rate (Table 1).

We also found that datasets that displayed a significant correlation between interactions and evolutionary rate, also displayed similarly strong correlations between abundance and numbers of interactions. The correlation between abundance and interactions was positive, i.e. proteins with high expression levels would participate in more interactions (Table 1).

Partial correlations for evolutionary rate and connectivity when protein abundance is controlled for were also calculated (Table 1). Controlling for protein abundance reduced the magnitude of any significant correlation between evolutionary rate and connectivity.

### Evolutionary excess retention

**Figure 5:**
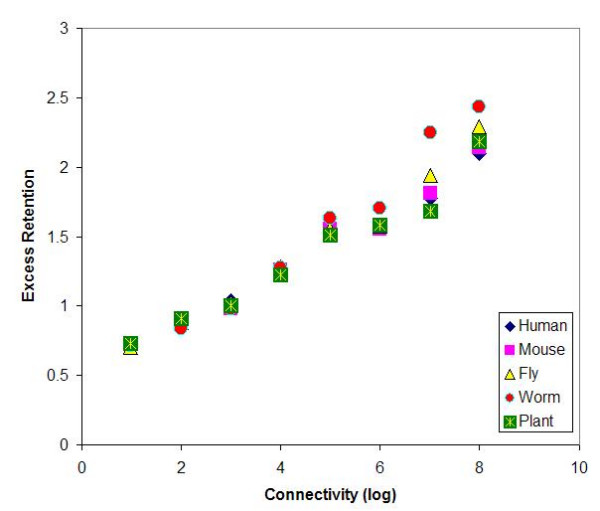
**Evolutionary Excess Retention and Connectivity**. The relationship between connectivity and excess retention (age) of a protein *ρ*: 0.98301. Proteins were binned on a log scale and the propensities of each bin possessing orthologs in several other species was calculated. Proteins that participate in more interactions tend to be older.

Figure 5 shows the relationship between ER and protein connectivity. The proteins have been logarithmically binned by their number of interactions. It can be seen that proteins with a high number of interactions tend to be older (have a higher ER) than proteins with fewer interactions.

**Figure 6:**
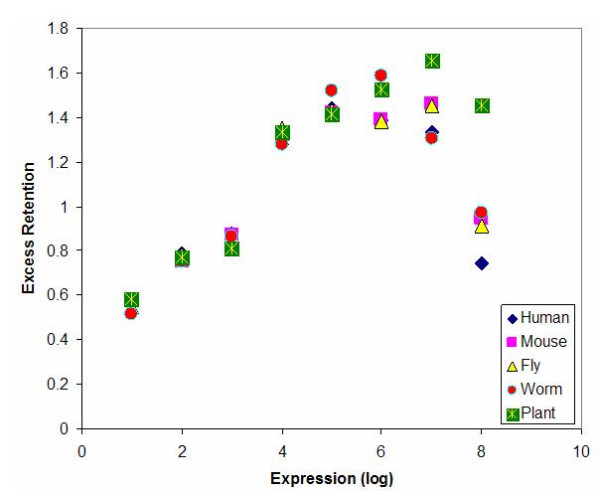
**Evolutionary Excess Retention and Abundance**. The relationship between the expression level of a protein and Excess Retention shows a strong correlation *ρ*: 0.67866. Proteins that are highly abundant in the cell tend to be older.

Figure 6 shows the relationship between the expression levels of proteins and ER. The relationship is similar to that observed between ER and interactions, however the correlation is not as strong in this case.

## Discussion

### Connectivity vs evolutionary rate

In our analysis of several different datasets we found that a correlation between protein interactions and evolutionary rate exists in some datasets and not in others (Figure 2). Where correlations were observed, they were weak, (between Spearman's *ρ*: -0.1 and -0.25)) but statistically significant (P-value: < 10^-3^). The weakness of observed correlations could be attributed to the incomplete nature of the known yeast interactome. It has been estimated that the ~6000 proteins in yeast participate in at most 40,000 interactions [38-40]. GRID, the largest dataset contains 4907 proteins and a total of 17,598 interactions. Considering this also includes a percentage of false positives, it is clear that only a fraction of yeast interactions have been measured. There is also very little overlap between interactions returned from different experimental methods. Results returned by Gavin et al (2002) measure 3957 interactions using the TAP method. Only 63 of these interactions can be found in the Uetz et al's (2000) dataset. Although this could be due to different experimental methods favouring different types of interactions, the lack of agreement denotes the partial nature of the picture to date.

The large fraction of missing data on evolutionary distance is another factor that may explain the weakness of correlations. When searching for orthologs in the *M musculus *we found BRH orthologs for only half of the proteins in the GRID dataset, (Table 1). This resulted in 50% of the nodes from the interactome missing from our final correlation graph (Figure 1). A highly interacting node which is missing one interaction would simply move its position on the graph, however if the node itself is missing the graph will be missing a point. In this case 50% of the points are missing and it is entirely possible that the 50% that are present may have the wrong number of interactions.

To address the issue of missing orthologs, we searched for orthologous proteins in the more closely related species *S paradoxus*. This resulted in far more orthologs being found (over 80%), however the strength and general pattern of the correlations remained the same as before (Figure 2). The slight disparity between the *cerevisiae-musculus *and *cerevisiae-paradoxus *based correlations is probably due to the relatively small amount of evolutionary change that occurred between the *cerevisiae *and *paradoxus*. However the correlation still remains weak even though we located more orthologs. It therefore seems that the primary reason for the weak magnitude of the correlation may be the incomplete nature of the network. Our error rate analysis showed that the accuracy of sets varied (Table 2). When considering the accuracy of the sets it is important that we consider the three error rate indicators collectively. Considering any error rate indicator on its own could be misleading as the experimental methods used could bias a particular error rate indicator. For example the MIPS_Genetic dataset has a high EPR index (74.9%). This indicator on its own suggests that this dataset is highly accurate. However the Reference index is 4.08% and the LS index is 27.52%. The EPR index value is explained by the experimental methods used to obtain the interactions in this dataset. The experimental methods used (synthetic lethality and suppression analysis) check for functional interactions. Functionally related genes tend to be expressed in a similar manner and so a high EPR index would be expected.

In order to translate error rates into a meaningful consensus based representation, we calculated the average rank over the three independent measures to give us an Average Accuracy Rank (Figure 3). Three datasets have been omitted. The MIPS_GENETIC dataset as it is a set of functional interactions rather than physical interactions. The BIND dataset as we could only calculate one of the three error rates for it, and the INTACT_SMALL dataset as it will give uncertain error rates due to its small size. From Table 2 and Figure 3, it is clear that the MIPS_PHYSICAL dataset shows no correlation and its accuracy level is amongst the lowest. In general we find that datasets that demonstrate stronger correlations between connectivity and evolutionary rate are more accurate whereas datasets that show no correlation are found to be less accurate.

The UETZ dataset has relatively high accuracy levels for all three of our error measures. Its consensus accuracy is higher than that shown by GRID and MINT (Figure 3). However it shows no statistical correlation between connectivity and rate of change. The UETZ dataset is obtained via the Y2H method. This has previously been shown to be an inaccurate experimental process [13]. A plausible explanation for the lack of correlation is its lack of representation of highly interacting proteins. The UETZ dataset contains a comparable number of proteins to the GAVIN and HO sets, yet it contains a significantly lower number of interactions (Table 1). The UETZ dataset contains 1438 interactions for 1328 proteins which averages to no more than 1.08 interactions per protein, whereas the GAVIN and HO sets average over 2.3 interactions per protein. It is fair to say that with just 1.08 interactions per protein, proteins with more than one interactions are highly under-represented in the UETZ dataset.

The HO dataset has a low accuracy according to our consensus measure (Figure 3), yet it returns a strong-correlation. It possessed the lowest EPR Index and LS index from amongst all the datasets. Von Mering's analysis estimated the experimental method used in this set to have an accuracy of only 2% [13]. The strength of the correlation between connectivity and evolutionary rate in this dataset could be due to a previously discussed artificatually generated association between connectivity and expression level [9]. Specifically the artificial correlation shown by the HO dataset could arise from the experimental method used to generate the interactions. Ho et al used the HMS_PCI protocol, where the bait proteins are transiently overexpressed. This overexpression may have led to the detection of a large number of false interactions for highly expressed genes. However our partial correlation analysis does not support this conclusion, as we find that if we control for expression level, the correlation observed between connectivity and evolutionary rate still exists.

An analysis of the overlap between the accumulative datasets (sets containing data from many sources) and single experimental method datasets (HO, GAVIN, ITO, UETZ) further corroborates findings from the error rate analysis (Table 3). The DIP_Core dataset, has very little overlap with the inaccurate ITO dataset. This gives further support to our initial assertion that the DIP_Core dataset contains a large fraction of good interactions. Interestingly the three accumulative datasets, BIND, MIPS_Genetic and MIPS_Physical, which showed no correlation between interaction and connectivity, had very little overlap with the GAVIN dataset. The GAVIN dataset is obtained by the affinity purification method and our error rate analysis considers it to be quite accurate. The BIND and MIPS databases are missing a very large fraction of affinity-purification data.

It was also noted that the MIPS_Genetic dataset has very little overlap with any of the single experimental method datasets. To a certain extent this is to be expected as the MIPS_Genetic set contains functional interactions as opposed to physical interactions. The lack of congruence between the MIPS_Genetic set and physical interaction datasets highlights the stark differences between functional interactions and physical interactions.

### Abundance vs evolutionary rate

We used the mRNA expression levels in yeast as a measure of abundance. Table 1 shows the correlations observed in all the datasets, between the three factors, evolutionary rate, abundance and interactions. Strong and significant correlations between abundance and evolutionary rate are detected for all the datasets bar the SMALL dataset. A possible explanation for the absence of a correlation in the SMALL dataset is that within this set only 60 proteins had both evolutionary rate and expression information. As has previously been the case, such a small set of nodes may be lacking enough information to display a significant correlation.

Datasets with high accuracy also demonstrated a relationship between abundance and interactions. A positive correlation (where abundant proteins tend to possess more interactions), of a similar magnitude to that observed between interactions and evolutionary rate was seen. This correlation was not observed in sets which were considered to be inaccurate. A simple explanation for this could be that proteins which are broadly present in the cell will have a greater functional role and therefore will participate in many interactions.

When comparing the two correlations, the strength of the abundance vs. evolutionary rate correlation is far stronger than the correlation of interactions vs. evolutionary rate. An explanation for this could be that the interactome is far from complete as discussed earlier. Expression data on the other hand, is far more exhaustive, with expression levels known for 6172 proteins [34]. Expression data is also thought to be of a better quality [15].

Previously it was suggested that affinity-purification methods were biased in that they measured more interactions for highly expressed proteins [9]. This assertion was based on the observation, only in affinity purification sets, of a positive correlation between number of interactions and expression levels, i.e. highly expressed proteins had more interactions. It is a questionable claim, as it can be said that highly expressed proteins are more abundant because of their important functional role, and such a role may require it to interact with many proteins.

Our findings throw further doubt on the claim as we also observed positive correlations between expression levels and number of interactions in accumulative datasets as well as our "golden standard" dataset DIP_Core. Accumulative datasets contain interaction information from different sources, small scale, Y2H and TAP methods. The DIP_Core dataset, our dataset of true interactions, compiled and verified from different sources shows a significant positive correlation between expression and interactions (Spearman's *ρ*:0.1755).

Nevertheless Bloom et al went on to conclude that because of the stronger positive correlation between expression levels and evolutionary rate, the expression levels were responsible for any correlation between the number of interactions and the evolutionary rate.

To judge what effect, if any, expression levels had on the relationship between connectivity and evolutionary rate we calculated the partial correlations based on the Spearman's rank correlation. When controlling for expression, we found that the strength of the correlation between interactions and evolutionary rate did decrease slightly, in cases where it was observed in the first instance (Table 1). This however does not imply that expression levels are the reason why we observe a correlation between interactions and rate of change. Judging by the strength of the correlation between expression and evolutionary rate, we believe that expression is simply a better predictor of evolutionary rate than connectivity.

### Interactions vs age

We found proteins with a high ER value tend to participate in more interactions. Conducting our analysis on the DIP_Core dataset, we found a very strong correlation between ER and interactions (Figure 5). This supports the belief that hub proteins are more likely to be older than non-hub proteins and corroborates previous work [41,42]. The ER differential can be explained by the theory of preferential attachment [43]. New protein's once having entered the interactome by a growth process, are more likely to form connections with proteins that are already highly interacting. As a result of this process proteins that are present in the interactome for a longer period will accrue more interactions, i.e. hub proteins are older. The scale-free nature of the interaction network could also be explained by such a growth process [44]. We also examined the relationship between ER and expression. This correlation was slightly weaker than the correlation between ER and interactions, yet still significant (Figure 6). The correlation suggests that proteins that tend to be older are more abundant. Collectively this suggests that older proteins are not only highly expressed but also participate in more interactions.

**Figure 7:**
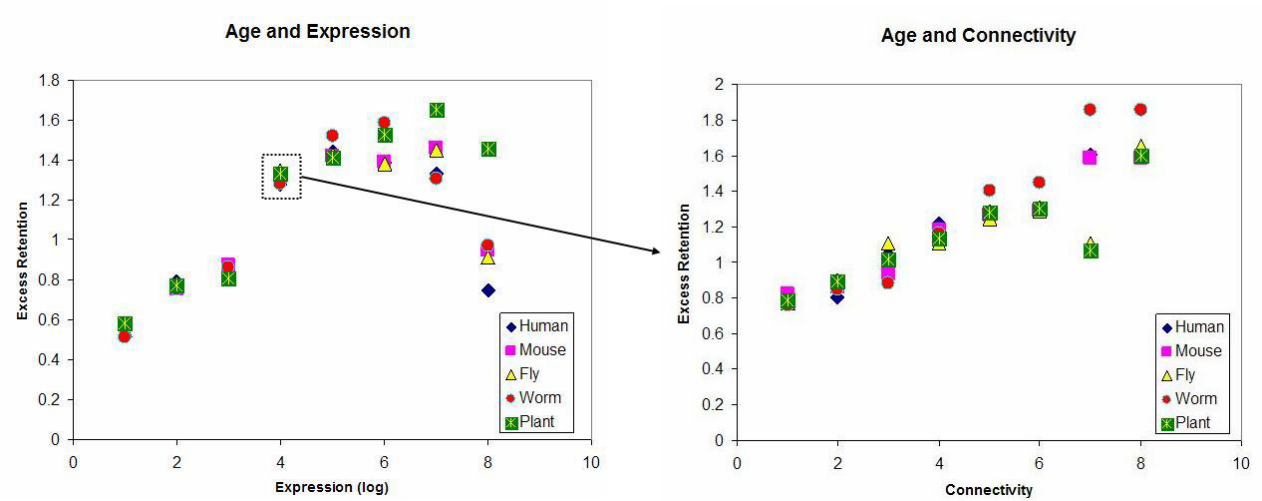
**Age and Connectivity when controlling for Expression**. To control for expression, a bin of proteins is selected from the Age and Expression graph. The proteins in this bin are reanalysed to check if the correlation between age and connectivity still remains. This stratification demonstrates that expression levels have very little effect on the relationship we observe between age and interactions *ρ*: 0.91762).

In order to ensure that expression was not causing a bias in the relationship between ER and interactions, we took all the proteins from several expression bins and checked for an association between ER and interactions. This stratification analysis is an effective way of checking if expression has any affect on the relationship between ER and connectivity (Figure 7). For the DIP_Core dataset a correlation, between ER and number of interactions, of strength *ρ*: 0.98301 was detected, this correlation remained when we examined an expression based bin in which a large number of proteins (> 50) were present (*rho *: 0.91762). This indicates that the abundance levels of proteins had very little effect on the relationship between ER and interactions.

## Conclusion

The relationship between protein connectivity and rate of change has been unclear for some time. We aimed to clarify the issue by studying various datasets and factoring for error rates within sets. We also analysed what impact the number of interactions had on other attributes of proteins such as abundance levels and protein age. We have shown that the relationship between interactions and evolutionary rate does exist, confirming that proteins with more interactions change less. We attribute the weakness of the correlation to the incomplete nature of the interactome rather than the number of orthologs found. Our error rate analysis has shown that datasets with low accuracy do not show any correlation while high accuracy sets display a correlation.

In line with earlier findings we also found that a strong association between abundance and interactions exists. Proteins that are highly abundant in the cell participate in more interactions. Interestingly interaction sets that were of high accuracy, also showed a positive correlation between abundance and interactions. Controlling for abundance shows a reduction in the magnitude of the correlation between interactions and evolutionary rate. The strength of the correlation between expression and evolutionary rate suggests that expression is a better predictor of evolutionary rate than connectivity.

The age of a protein, as represented by ER, has a very strong and significant relationship with protein connectivity. The older a protein is the more interactions it has. This is possibly explained by the theory of preferential attachment. Older proteins were also found to be more abundant, further strengthening the relationship we found between abundance and number of interactions. However controlling for abundance does not significantly weaken the relationship between age and interactions. This strongly suggests that the theory of preferential attachment in interaction networks is correct.

## Figures and Tables

**Table 1:** Datasets and their correlations. The different datasets that were used in the study and the number of their constituent proteins and interactions. The total number of proteins is shown, as well as the number of proteins for which sequence information was obtainable and the number of orthologs found in the *Mus musculus*. The final four columns show the correlations between three factors, Evolutionary Distance (ED) as measured using Grishin's method, Abundance (A) and Number of interactions (I). P-values for these correlations were calculated, values in bold have a p-value greater than 0.03. The final column shows the result of a partial correlation, between evolutionary rate and number of interactions where abundance has been controlled for. The BIND datasets lacked expression information due to nomenclature issues.

Dataset	Number of Proteins			Number of Interactions	ED vs I	ED vs A	A vs I	ED vs I. controled A
						
	Total	Sequences	Orthologs					
MIPS_GENETIC	1628	1618	903	5113	**0.005**	-0.4381	**0.018**	0.015
MIPS_PHYSICAL	4154	4124	2064	7458	**-0.017**	-0.410	**-0.064**	-0.048
BIND	4542	3999	2084	8649	**-0.032**	-	-	-
GRID	4907	4897	2381	17598	-0.118	-0.416	0.135	-0.069
MINT	4595	4590	2269	12582	-0.143	-0.407	0.174	-0.080
DIP_FULL	4773	4772	2354	15481	-0.121	-0.411	0.164	-0.059
DIP_CORE	2640	2640	1552	6600	-0.168	-0.397	0.176	-0.109
INTACT_UETZ	1328	1325	816	1438	**-0.077**	-0.391	**-0.045**	-0.103
INTACT_ITO	3245	3241	1675	4449	**0.030**	-0.4172	**0.001**	0.033
INTACT_GAVIN	1471	1467	1014	3957	-0.172	-0.431	0.172	-0.109
INTACT_SMALL	94	92	63	134	**-0.208**	**-0.087**	-0.122	-0.221
INTACT_HO	1577	1573	1036	3618	-0.227	-0.388	0.213	-0.160

**Table 2:** Error Rate. The EPR index is an estimate of the percentage of true positives in the set. The Reference Index is the percentage of proteins from the reference set found to be in the set of interest. The LS index is the percentage of interactions where both interacting partners shared the same subcellular localisation. The correlation observed between the Evolutionary rate (ED) and the number of Interactions (I) is shown. Values in bold type had a P-value greater than 0.03

Dataset	EPR Index (%)	Reference Index (%)	Localisation Similarity (%)	ED vs I
INTACT_ITO	15.7	13.59	33.33	0.0300
MIPS_GENETIC	74.9	4.08	27.52	0.0051
MIPS_PHYSICAL	55.3	22.66	30.16	-0.0172
BIND	-	24.78	-	-0.0316
INTACT_UETZ	42.4	31.64	33.38	-0.0774
GRID	43.9	23.05	33.46	-0.1183
DIP_FULL	41.8	42.72	38.67	-0.1207
MINT	31.6	27.1	37.01	-0.1428
DIP_CORE	73.9	100	51.11	-0.1682
INTACT_GAVIN	74.1	48.62	60.58	-0.1715
INTACT_SMALL	196	29.57	71.64	-0.2081
INTACT_HO	3.78	28.89	24.23	-0.2270

**Table 3:** Overlap. The percentage overlap between single experimentally derived sets (HO, GAVIN, ITO, UETZ, SMALL) and compound datasets. Compound datasets are those sets that contain information from a range of different experimental sets.

Dataset	INTACT_HO	INTACT_GAVIN	INTACT_ITO	INTACT_UETZ	INTACT_SMALL
DIP_CORE	28.90	48.60	13.60	31.60	29.60
DIP_FULL	85.10	75.20	91.80	87.70	29.60
MINT	73.10	85.30	98.00	96.20	27.00
GRID	82.60	73.60	88.00	89.40	33.90
BIND	5.80	8.60	74.70	59.10	24.30
MIPS_PHYSICAL	4.60	8.00	86.70	61.80	20.00
MIPS_GENETIC	0.70	1.00	0.40	1.20	2.60
